# Precision Monitoring in a Sepsis Mimic: A Case Report of Idiopathic Capillary Leak Syndrome in the Surgical ICU

**DOI:** 10.3390/healthcare14182930

**Published:** 2026-09-09

**Authors:** Tina Tomić Mahečić, Vedran Premužić, Robert Baronica, Marija Trbojević, Lucija Biličić, Ivana Janja Bošnjak, Antonia Vukšić, Iva Tucić

**Affiliations:** 1 Department of Anaesthesiology and Intensive Care Medicine, University Hospital Centre Zagreb, 10000 Zagreb, Croatia; rbaronica@gmail.com (R.B.); bilicic.lucija@gmail.com (L.B.); ijanjabosnjak@gmail.com (I.J.B.); vuksic.antonia55@gmail.com (A.V.); ivatucic@gmail.com (I.T.); 2 Department of Internal Medicine, University Hospital Centre Zagreb, 10000 Zagreb, Croatia; vpremuzic@gmail.com; 3 Department of Anaesthesiology and Intensive Care Medicine, General Hospital Bjelovar, 43000 Bjelovar, Croatia; marijat1804@gmail.com

**Keywords:** idiopathic capillary leak syndrome, Clarkson’s disease, critical care, shock, immunology

## Abstract

**Highlights:**

**What are the main findings?**

- Hypotension, hemoconcentration, hypoalbuminemia.
- The diagnosis of ICLS remains clinical and is a diagnosis of exclusion.

**What are the implications of the main findings?**

- Importance of multidisciplinary management.
- Need for precision monitoring.

**Abstract:**

Idiopathic Capillary Leak Syndrome (ICLS), also known as Clarkson’s disease, is a rare yet potentially life-threatening disorder of unknown etiology. It is a clinical condition that is frequently overlooked, but represents a severe and potentially fatal illness, characterized by fluid extravasation into the interstitial space, resulting in hemoconcentration, generalized edema, effusions, and hypotension. Owing to the similarity of its symptoms to those of other conditions, ICLS is often underdiagnosed, thereby posing a challenge for patient management. We report a complex case of a 69-year-old patient who presented with shock secondary to a necrotic cecal tumor and was ultimately diagnosed with ICLS. This case underscores the diagnostic challenges, the need for awareness and need for precision monitoring in differentiating ICLS from other causes of shock and emphasizes the critical importance of early recognition and appropriate therapeutic intervention. Additionally, a review of the current understanding of ICLS pathophysiology, diagnostic criteria, and treatment strategies, based on existing evidence, is provided.

## 1. Introduction

Idiopathic capillary leak syndrome (ICLS), also known as Clarkson’s disease, is a rare and potentially life-threatening disorder characterized by recurrent episodes of acute plasma extravasation from the intravascular to interstitial compartments [1]. This transient, severe but reversible hemoconcentration and hypoalbuminemia is caused by leakage of fluids and macromolecules into tissues, leading to generalized edema and hypovolemic shock [1]. Clinically, these episodes are defined by a characteristic triad of profound hypotension, hemoconcentration, and hypoalbuminemia, requiring intensive care management [2].

Fewer than 500 cases have been documented in the medical literature since the condition’s initial description in 1960 [3]. Owing to its rarity and nonspecific presentation, ICLS is frequently underdiagnosed or misattributed to conditions such as culture-negative sepsis, isolated hypoalbuminemia, or distributive/hypovolemic shock of other etiologies and much of the existing clinical knowledge is derived from individual case reports [4].

Capillary leak syndrome may be classified as idiopathic or secondary, the latter occurring in association with conditions such as sepsis, major trauma, engraftment syndrome, or severe acute pancreatitis [5]. The diagnosis of ICLS remains clinical and is a diagnosis of exclusion.

Diagnosis of ICLS requires high clinical suspicion when there is a clinical triad: acute shock/hypotension, hemoconcentration, and hypoalbuminemia in the absence of other explanations (other causes of shock such as sepsis, anaphylaxis, cardiac causes, and bleeding), with laboratory workup showing a pathognomonic combination of hemoconcentration and hypoalbuminemia and identification of monoclonal gammopathy through serum and urine protein electrophoresis in adult patients [3].

The underlying pathophysiology of ICLS involves transient yet marked increases in endothelial permeability, leading to fluid and plasma protein leakage. Recent mechanistic studies have highlighted intrinsic endothelial dysfunction and heightened sensitivity to inflammatory mediators as central features of this disease [6]. Endothelial cells derived from patients with ICLS demonstrate exaggerated responses to vascular permeability factors such as vascular endothelial growth factor and histamine, which are partially mediated by increased nitric oxide synthase activity [6]. Although capillary hyperpermeability is typically reversible, delayed recognition and management may lead to severe complications and high mortality rates [7,8]. ICSL is a potentially fatal disorder, with reported mortality ranging from 9.8% per acute episode in a contemporary registry to 18.6% among severe episodes requiring ICU admission; historical cohorts have reported five-year survival rates of approximately 73–78% [3].

The acute management of ICLS is primarily supportive and requires careful, individualized hemodynamic optimization. Judicious intravascular volume expansion with crystalloid solutions and, in selected cases, synthetic colloids may be necessary to maintain organ perfusion; however, excessive fluid administration should be avoided, as it has been associated with increased complication severity, including rhabdomyolysis and the need for renal replacement therapy [3]. Additional supportive measures may include oxygen supplementation, vasoactive agents for hemodynamic stabilization, corticosteroids, and, in selected cases, theophylline [9]. During the acute phase, early administration of intravenous immunoglobulin (IVIG), particularly when initiated close to symptom onset and combined with additional intravenous fluid, has been associated with favorable clinical outcomes and rapid improvement [9].

Notably, more than 80% of patients with ICLS have an associated monoclonal gammopathy, typically IgG kappa type, suggesting a possible pathogenic link, although its precise role remains unclear [4].

Here, we report a case of idiopathic capillary leak syndrome with an unusual initial presentation of an acute abdomen, without proven sepsis, highlighting the diagnostic challenges and clinical variability of this rare disorder, with the aim of identifying clinical and laboratory parameters that may facilitate the early diagnosis of ICLS and increase awareness of this rare condition.

## 2. Case Presentation

A 69-year-old man with a medical history of chronic arterial hypertension was brought to the emergency department by the emergency medical services in March 2025 with progressive abdominal pain and profound hypotension. Abdominal pain had begun two days prior to admission and was accompanied by diarrhea, generalized weakness, anorexia, and reduced oral intake. The patient reported no fever, vomiting, or recent travel. At the time of presentation, the patient underwent outpatient gastroenterological evaluation for newly diagnosed ascites of unclear etiology.

On arrival, the patient was alert and oriented, but appeared acutely ill. Vital signs revealed severe hypotension (blood pressure, 70–80/40 mmHg) and tachycardia (heart rate, 115 beats/min) with a normal body temperature. Physical examination revealed diffuse abdominal tenderness with rebound guarding without focal peritonitis. Peripheral edema was absent at presentation.

**Figure 1:**
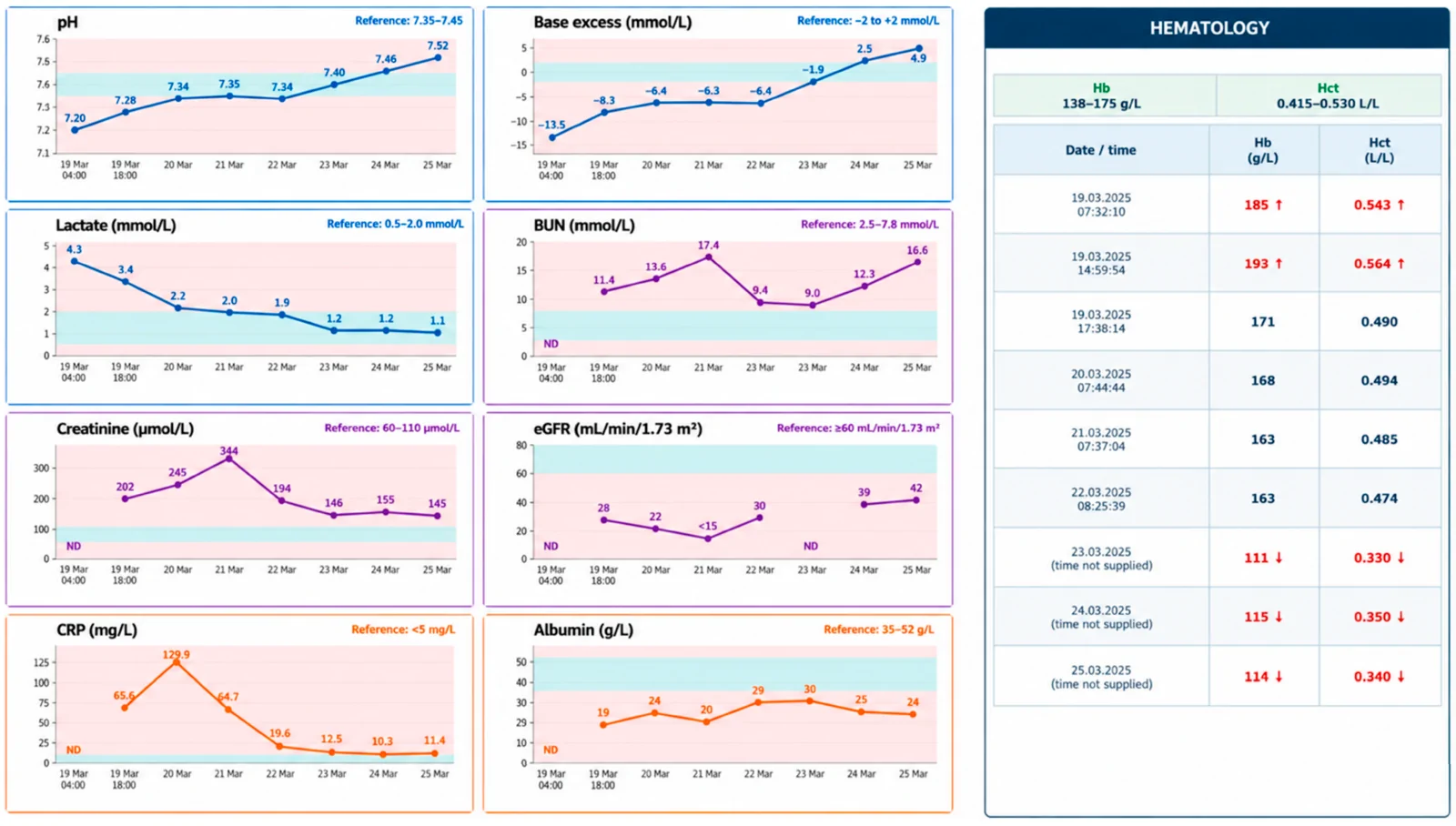
Laboratory findings during the first 6 days in the intensive care unit.

Initial laboratory investigations showed marked leukocytosis with a pronounced inflammatory response, severe hemoconcentration, and polycythemia (hematocrit 54,3%), acute kidney injury, hypoalbuminemia, and metabolic derangements (Figure 1).

Arterial blood gas analysis revealed high-anion-gap metabolic acidosis with lactic acidemia, consistent with global hypoperfusion. Inflammatory markers were markedly elevated (CRP 65.6 mg/L), and renal function was impaired, with elevated urea and creatinine levels. Serum protein and albumin concentrations were significantly reduced (Figure 1).

**Figure 2:**
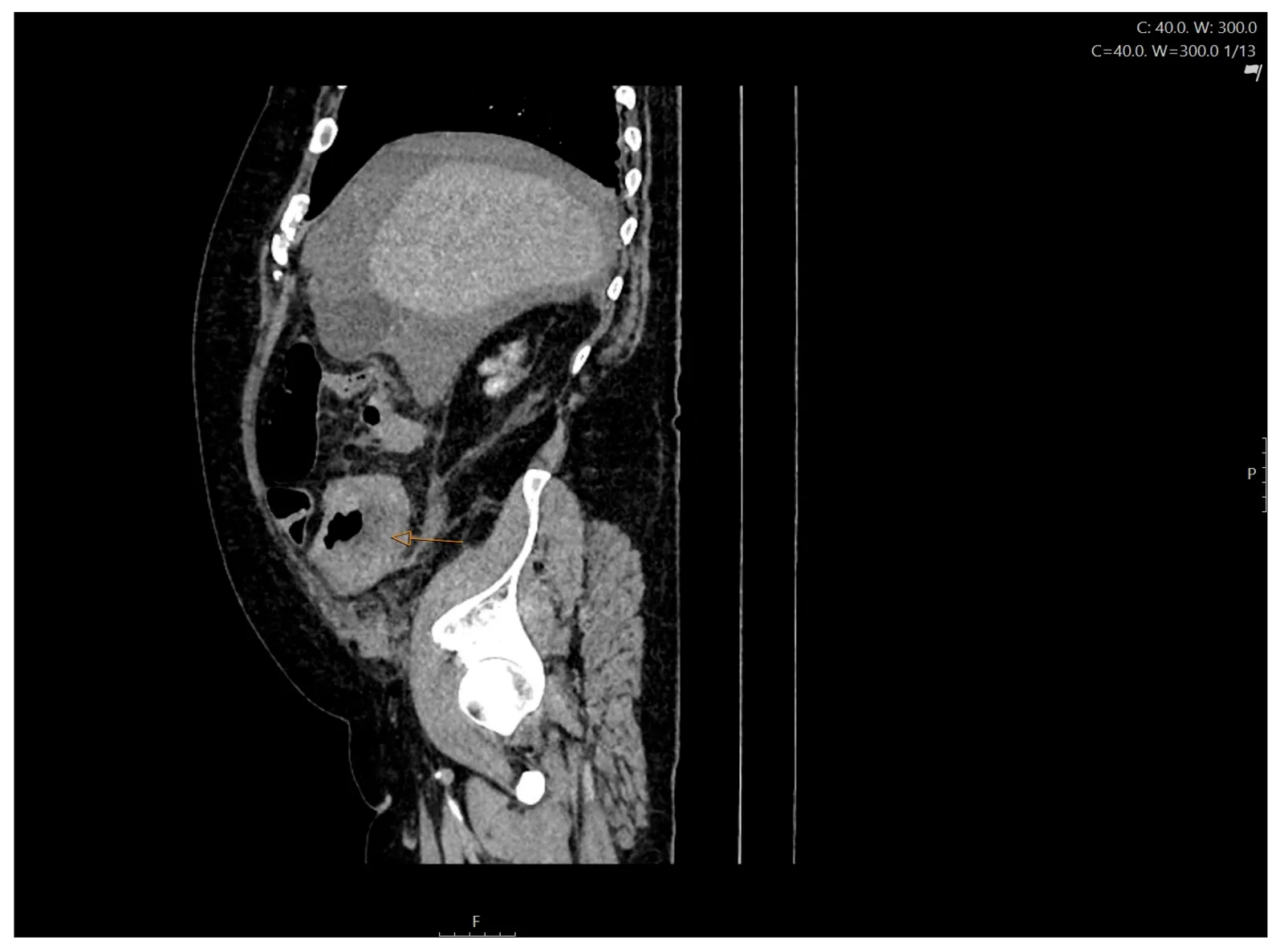
CT scan showing ascites and eccentric wall thickening of the cecum and terminal ileum with a large hypovascular area consistent with a necrotic tumor extending approximately 6 cm craniocaudally (yellow arrow).

Given the combination of acute abdomen and circulatory shock, contrast-enhanced computed tomography of the abdomen and pelvis was performed. Imaging showed eccentric wall thickening of the cecum and terminal ileum with a large hypovascular area consistent with a necrotic tumor extending approximately 6 cm craniocaudally (Figure 2).

Moderate ascites and bilateral pleural effusions were also present with no evidence of intra-abdominal abscess or free air. These findings were suggestive of locally advanced cecal malignancy complicated by shock, and an abdominal surgeon was urgently consulted.

The patient underwent an urgent right hemicolectomy. During the procedure, standard microbiology samples were taken and sent to the microbiology department. Intraoperatively, despite aggressive fluid resuscitation, persistent hypotension necessitated initiation of norepinephrine infusion. Postoperatively, he was transferred to the surgical intensive care unit (ICU) for ongoing management of refractory shock. Treatment was initiated in accordance with sepsis guidelines and included aggressive volume resuscitation with crystalloids, broad-spectrum antibiotics (piperacillin–tazobactam and vancomycin), and vasopressor support (norepinephrine).

**Figure 3:**
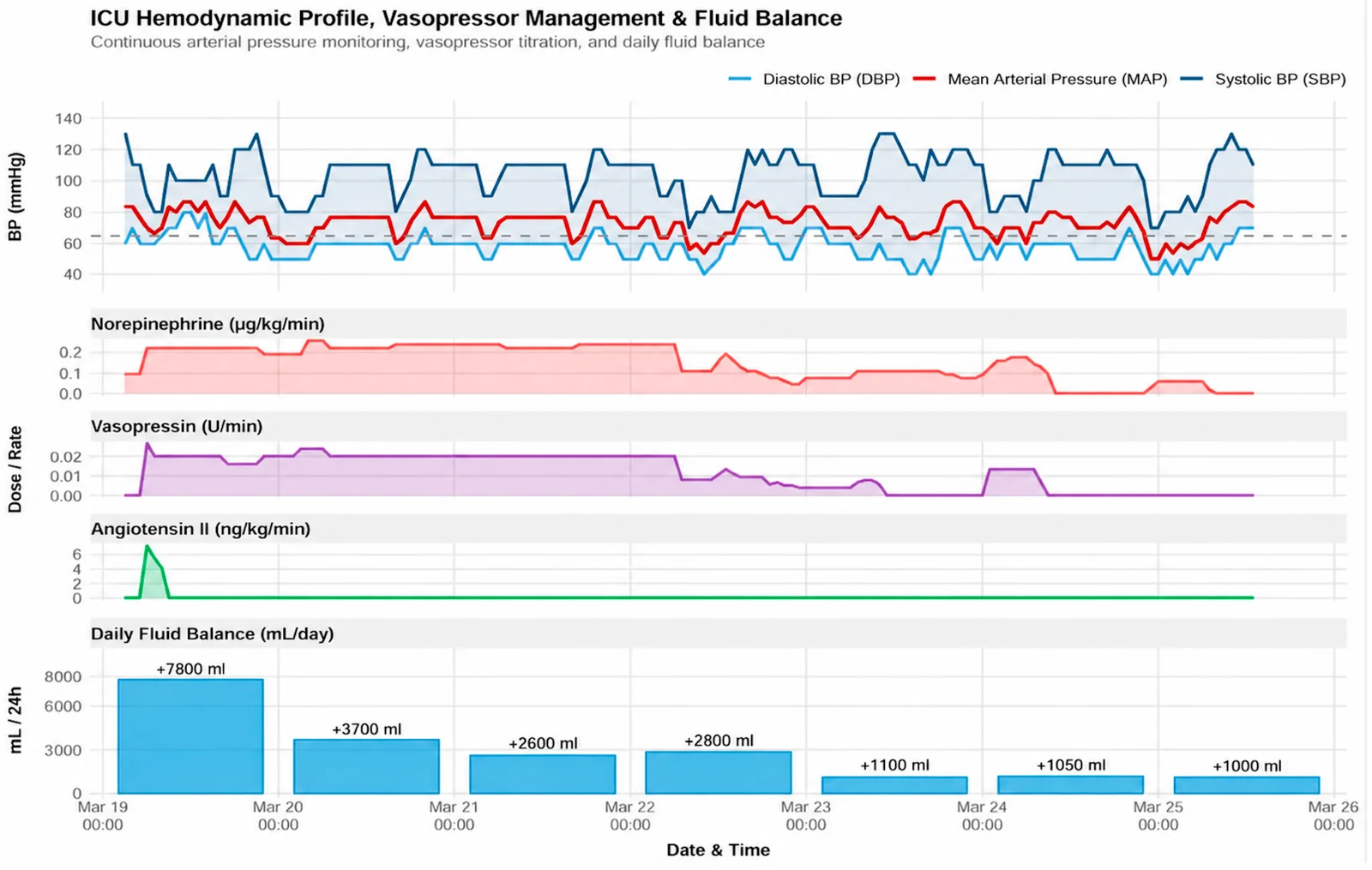
Hemodynamic course. Temporal evolution of fluid balance and vasopressor requirements during the acute and recovery phases of ICSL.

During the first 24 postoperative hours, the patient required exceptionally high-volume fluid resuscitation, receiving more than 13 litres of crystalloids in addition to albumin supplementation. Nevertheless, hemodynamic instability persisted and escalating vasoactive therapy with norepinephrine, vasopressin, and angiotensin II was required (Figure 3).

Advanced hemodynamic monitoring (FloTrack system by Edwards Lifesciences) demonstrated a low cardiac output state, with a reduced cardiac index (CI 1.7 L/min/m^2^) and a severely decreased stroke volume index (SVI 17 mL/m^2^). Stroke volume variation was markedly elevated (SVV 22%), indicating significant preload dependence and suggesting potential fluid responsiveness. The systemic vascular resistance index was increased (SVRI 2848 dyn·s·m^2^/cm^5^), consistent with an elevated afterload, likely due to vasopressor therapy. Focused transesophageal echocardiography showed preserved left ventricular systolic function with normal contractility, but underfilled cardiac chambers and a collapsible inferior vena cava, with no evidence of right ventricular dysfunction. Overall, these findings were consistent with a low-output, preload-dependent circulatory state characterized by increased systemic vascular resistance.

Despite continued fluid administration, the patient developed progressive hemoconcentration and worsened hypoalbuminemia. Massive fluid loss became evident, with high-volume serous output from abdominal drains exceeding 4 litres and rapid accumulation of bilateral pleural effusions, requiring placement of a left-sided thoracic drain, from which 1.5 litres of fluid was evacuated. Subsequently, generalized edema developed. Renal function deteriorated further with persistent oliguria despite diuretic therapy, prompting the initiation of continuous renal replacement therapy in consultation with nephrology. Plasmapheresis was performed as a therapeutic option to remove circulating mediators responsible for endothelial dysfunction.

**Figure 4:**
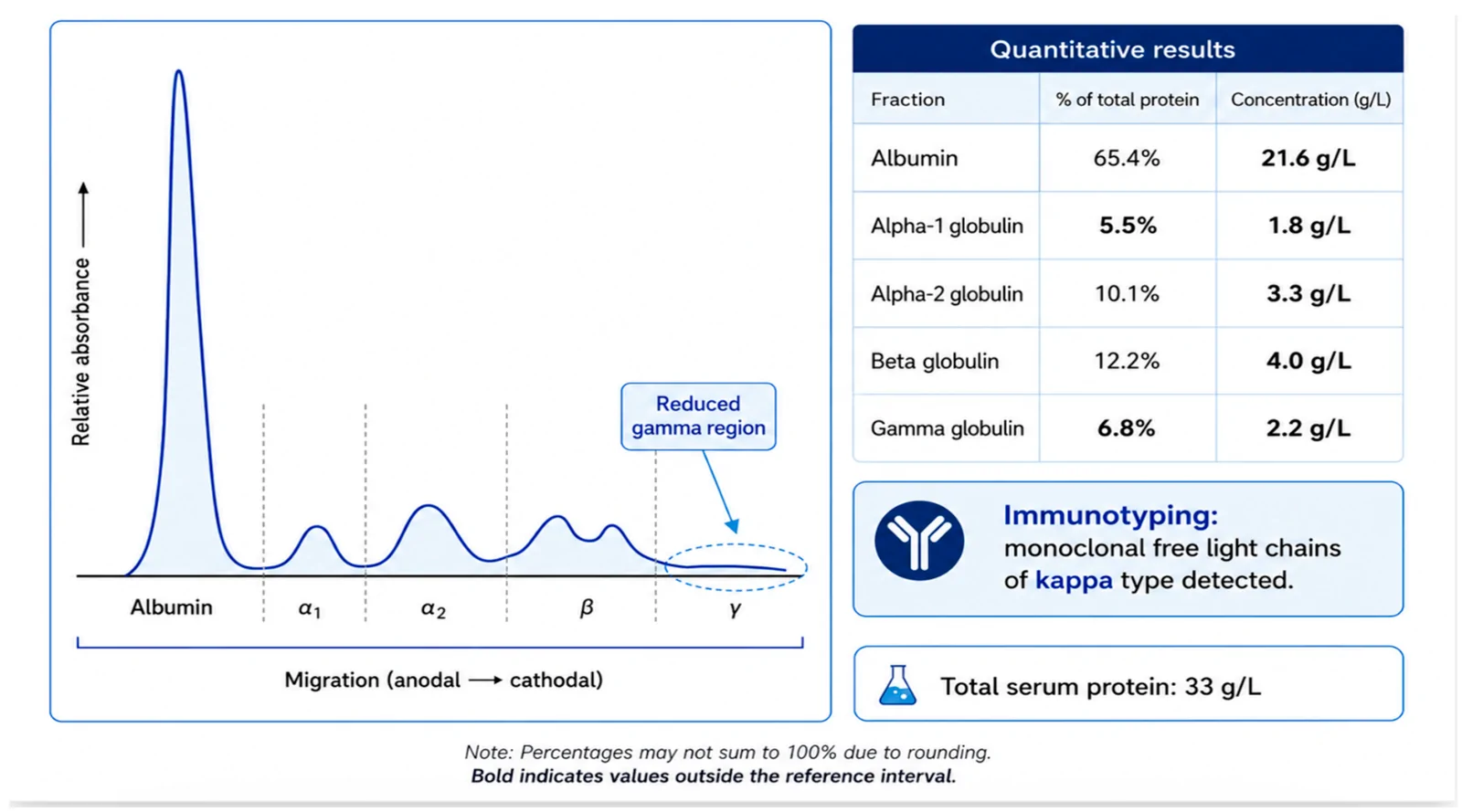
Serum protein electrophoresis and immunofixation.

Extensive diagnostic evaluation was performed to exclude sepsis and identify ongoing alternative causes of shock. All targeted investigations, blood and urine cultures, and repeat imaging failed to reveal the source of infection or intra-abdominal collection. Nephrotic syndrome was excluded based on clinical and laboratory criteria. The coexistence of refractory hypotension, hemoconcentration, profound hypoalbuminemia, and generalized edema despite aggressive fluid resuscitation raised a strong suspicion of idiopathic capillary leak syndrome. Further laboratory workup revealed marked hypoproteinemia, proteinuria, albuminuria, and erythrocytosis. A bone marrow biopsy showed proliferative atypical plasma cells, consistent with monoclonal gammopathy. Serum protein electrophoresis demonstrated increased free light chains with a pathological kappa-to-lambda ratio, raising concerns for monoclonal gammopathy-associated idiopathic capillary leak syndrome (Figure 4).

Treatment with intravenous immunoglobulins was initiated for three consecutive days.

Given the suspicion of multiple myeloma, a hematologist was consulted who recommended further diagnostic evaluation, including bone marrow aspiration with immunophenotyping and cytological analysis, bone marrow karyotyping, and fluorescence in situ hybridization (FISH) analysis. The requested workup was completed, during which a decline in serum free light chain levels was observed and no monoclonal protein was detected by immunofixation. Additionally, bone marrow immunophenotyping did not reveal any monoclonal B cells. These investigations did not demonstrate evidence of multiple myeloma or clonal plasma cell disorder, and the findings were ultimately interpreted as reactive.

**Figure 5:**
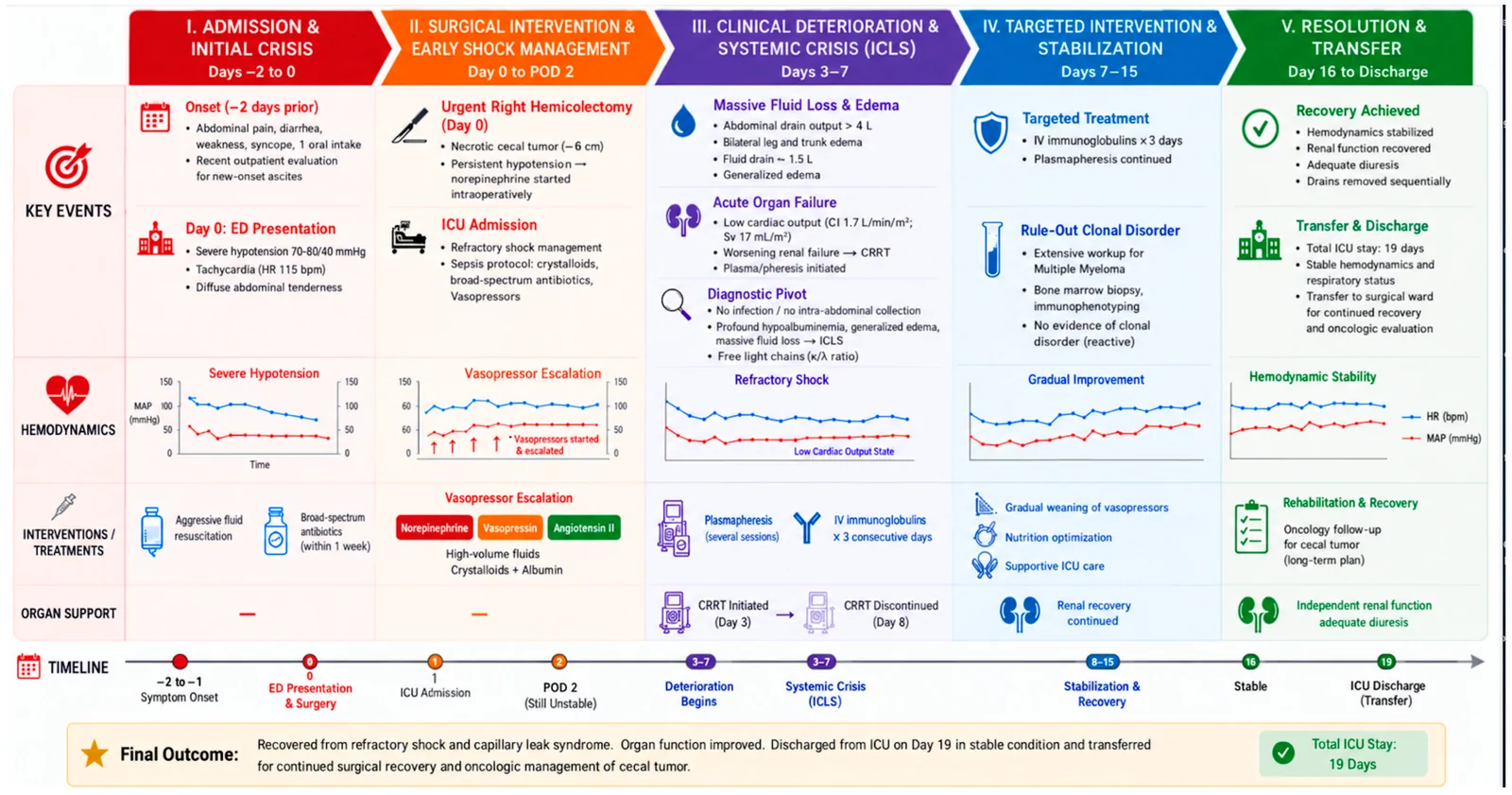
Timeline and clinical course.

Over the subsequent days, the patient’s hemodynamic status gradually improved, allowing de-escalation of the vasoactive support. Renal function recovered, and renal replacement therapy was discontinued on day 6 of admission. Fluid loss diminished, and pleural and abdominal drains were removed sequentially (Figure 5). After a total ICU stay of 19 days, the patient was discharged in a stable hemodynamic and respiratory state, with preserved renal function and adequate diuresis. The patient was transferred to the surgical ward for further recovery, oncological evaluation, and management of the cecal tumor.

## 3. Discussion

Idiopathic capillary leak syndrome (ICLS) is a rare but life-threatening disorder characterized by transient, profound increases in capillary permeability, resulting in extravasation of protein-rich plasma from the intravascular to the interstitial compartment [10].

Regardless of the etiology, all forms of capillary leak syndrome (CLS) share a common pathophysiological hallmark: endothelial barrier dysfunction leading to abrupt intravascular volume depletion, hemoconcentration, and hypoalbuminemia. The ensuing reduction in adequate circulating volume activates compensatory neurohumoral mechanisms, including the renin–angiotensin–aldosterone system, sympathetic nervous system, and vasopressin release, promoting sodium and water retention, generalized edema, ascites, and serous effusions [11,12].

Hypercytokinemia is thought to play a central role in this process by directly disrupting endothelial junctions and amplifying vascular permeability [12]. In severe acute episodes, rapid plasma loss leads to marked hemoconcentration, which is associated with disease severity and adverse outcomes [13,14].

Within this pathophysiological framework, the patient’s clinical course, characterized by refractory hypotension, progressive hemoconcentration, profound hypoalbuminemia, and massive third-space fluid accumulation, was highly consistent with an acute capillary leak episode.

Diagnostic Challenges and Differential Diagnosis

Diagnosing ICLS is notoriously difficult because its clinical presentation overlaps substantially with that of more common critical illnesses, such as septic shock, anaphylaxis, acute heart failure, and distributive shock of other etiologies [15].

In the present case, the initial presentation mimicked an acute abdomen complicated by septic shock. However, several features are atypical for classical sepsis, including the absence of fever, persistently negative microbiological cultures, disproportionate hemoconcentration despite massive fluid resuscitation, and severe hypoalbuminemia without evidence of hepatic failure or nephrotic syndrome.

The presence of solid tumors raises the possibility of secondary CLS triggered by malignancy-associated inflammation [4], but ICLS is most frequently associated with monoclonal gammopathy (80%) [3].

The diagnosis of ICLS remains clinical and is a diagnosis of exclusion.

In our patient, an extensive diagnostic evaluation excluded ongoing infection, cardiogenic shock, autoimmune disease, and primary renal pathology. Despite transient abnormalities in serum free light chains, a comprehensive hematological workup ultimately failed to demonstrate an underlying plasma cell dyscrasia, supporting the diagnosis of idiopathic systemic capillary leak syndrome (ICLS).

Clinical Phases of ICLS

ICLS typically follows a triphasic clinical course comprising three distinct but overlapping phases, each with unique pathophysiological features and therapeutic implications [4].

The *prodromal phase* lasts 1–2 days with nonspecific symptoms such as malaise, abdominal pain, and fatigue. Our patient’s days of discomfort and weakness likely matched this finding. The *extravasation phase* is critical and is marked by a sudden plasma leak causing severe hypotension, hemoconcentration, hypoalbuminemia, metabolic acidosis, oliguria, and rapid edema. Our patient needed over 13 litres in 24 h, with persistent hypotension and rising hematocrit, indicating severe leak and the limits of standard shock treatment. The *recovery phase* involves endothelial repair and fluid returning to blood vessels, but it increases the risks of pulmonary edema, compartment syndrome, and heart failure if fluids are mismanaged. Anticipating this phase and precision monitoring is vital to prevent complications.

Therapeutic Considerations

The management of ICLS remains supportive, mainly guided by clinical experience rather than high-quality evidence.

During the acute leak phase, cautious intravenous fluid resuscitation is required to preserve vital organ perfusion. However, unlike septic shock, excessive crystalloid administration may exacerbate interstitial edema and worsen outcomes during the recovery phase [5].

In our patient, persistent hypotension with only transient responses to volume therapy, despite preserved cardiac function, was an early clue for an alternative shock mechanism driven by capillary leakage rather than intravascular depletion alone. Vasoactive agents are often necessary to maintain adequate mean arterial pressure while minimizing fluid overload [15]. Advanced hemodynamic monitoring and echocardiography are invaluable for differentiating ICLS from cardiogenic shock and guiding individualized therapy.

Several pharmacological therapies have been proposed for ICLS, including corticosteroids, β-agonists, theophylline, and intravenous immunoglobulin (IVIG). Among these, IVIG has emerged as the most promising intervention [16,17]. Theophylline, terbutaline and verapamil are not first-line therapies and should only be discussed when IVIg are not available (in low-income countries) or in patients refractory to IVIg [10,18].

In our patient, early administration of IgM-enriched IVIG (Pentaglobin^®^) was based on a presumed immune-mediated mechanism stabilizing endothelial function and coincided with gradual hemodynamic stabilization and biochemical improvement.

Plasmapheresis has also been reported as a therapeutic option for severe or refractory cases aimed at removing circulating mediators responsible for endothelial dysfunction [9].

Supportive measures including renal replacement therapy, thromboprophylaxis, infection prevention, and meticulous fluid and electrolyte management remain essential components of care.

Diagnostic Criteria and Clinical Implications

The diagnosis of ICLS remains clinical. The classic diagnostic triad of hypotension, hemoconcentration, and hypoalbuminemia remains central to diagnosis [19]. Additional supportive features include episodic presentation, generalized edema, elevated hematocrit (>50%), low serum albumin (<25 g/L), and frequent association with monoclonal gammopathy reported in up to 80% of cases [4]. Crucially, alternative causes, such as infection, autoimmune disease, or primary malignancy-related shock, must be rigorously excluded.

## 4. Conclusions

This case emphasizes the importance of precision monitoring in ICU and considering idiopathic capillary leak syndrome (ICLS) in patients with edema, hypotension, and hemoconcentration, particularly when common infectious causes are ruled out. Other conditions may appear similar and may require different treatments. Early recognition is crucial for avoiding unnecessary interventions and reducing morbidity. Compared with standard approaches focused primarily on distributive shock management, this case emphasizes the need to recognize the physiology of idiopathic capillary leak early and adapt treatment accordingly, balancing organ perfusion against the risk of fluid overload to improve clinical outcomes.

The management of ICLS depends on its phase; therefore, physicians treating critically ill patients should understand its course. The idiopathic nature of this syndrome underscores the need for clinical vigilance and reporting of rare cases to improve awareness and guide management.

This case also illustrates the diagnostic and therapeutic challenges of idiopathic capillary leak syndrome (ICLS), particularly when it occurs alongside surgical issues and cancer.

Our report contributes to the existing literature by demonstrating the value of precision hemodynamic monitoring, early multidisciplinary collaboration, and timely consideration of targeted therapies such as IVIG.

## Data Availability

The original contributions presented in this study are included in the article. Further inquiries can be directed to the corresponding author.

## Abbreviations

The following abbreviations are used in this manuscript:

CI	Cardiac Index
CLS	Capillary Leak Syndrome
FISH	Fluorescence In Situ Hybridization
ICLS	Idiopathic Capillary Leak Syndrome
ICU	Intensive Care Unit
IVIG	Intravenous Immunoglobulin
SVI	Stroke Volume Index
SVV	Stroke Volume Variation
SVRI	Systemic Vascular Resistance Index
